# Bullying victimization and physical fighting among Venezuelan adolescents in Barinas: results from the Global School-Based Health Survey 2003

**DOI:** 10.1186/1824-7288-35-38

**Published:** 2009-11-25

**Authors:** Adamson S Muula, Patricia Herring, Seter Siziya, Emmanuel Rudatsikira

**Affiliations:** 1 Department of Public Health, Division of Community Health, University of Malawi, College of Medicine, Blantyre, Malawi; 2 Department of Health Promotion and Education, Loma Linda University, School of Public Health, Loma Linda, California, USA; 3 Department of Community Medicine, University of Zambia Medical School, Lusaka, Zambia; 4 Department of Epidemiology and Biostatistics, Loma Linda University, School of Public Health, Loma, Linda, California, USA; 5 Department of Global Health, Loma Linda University, School of Public Health, Loma Linda, California, USA

## Abstract

**Background:**

Violence among adolescents has untoward psycho-social and physical health effects among this age group. Most of the literature on this topic has been from high-income nations, and little information has come from middle- and low-income nations. This study was done to assess the relationship between physical fighting and bullying victimization among Venezuelan school-going adolescents in Barinas.

**Method:**

We used data from the 2003 Global School-Based Health Survey conducted among in-school adolescents in Barinas, Venezuela. We estimated the prevalence of bullying victimization and physical fighting. We also conducted Logistic regression analysis to assess the association between a selected list of explanatory variables and physical fighting. We hypothesized that there would be a dose-response relationship between physical fighting and number of times the adolescent reported being a bullied in the past 30 days.

**Results:**

A total of 2,249 adolescent students participated in the survey. However data on sex (gender) were available for only 2,229 respondents, of whom 31.2 (47.4% males and 17.0% females) reported having been involved in a physical fight in the last 12 months. Some 31.5% (37.0% males and 27.0% females) reported having been bullied in the past 30 days. There was a dose-response relationship between bullying victimization and physical fighting (p-trend < 0.001). Compared to subjects who were not bullied, those who reported being bullied were more likely to engage in physical fighting after controlling for age, sex, substance use (smoking, alcohol drinking or drug use), and parental supervision.

**Conclusion:**

Physical fighting and bullying victimization experience is common among in-school adolescents in Barinas, Venezuela. The fact that victims of bullying were more likely to have engaged in physical fighting may be evidence supporting the notion that "violence begets more violence".

## Background

Adolescent psychopathology and physical health other than substance use, teen premarital sex and its squealae and bio-medical conditions have not received much attention outside of Europe and continental United States. In continental United States for example, violence against adolescents is a major cause of morbidity and mortality among this age group [1,2]. Violence occurring on school property is also a major cause of concern, resulting in injury to students, teachers and other staff [3,4].

Adolescent violent behaviors are not only of public health significance because of the injury that they may be associated with but also for its associated behaviors. Previous research has shown that adolescents who report being exposed to violent behavior are also likely to engage in other unhealthy behaviors such as premarital sexual intercourse, illicit drug use and truancy. Adolescents who previously had been victims of violence are also likely to bear weapons on school property; thus potentially contributing further to serious consequences of inter-personal violence [5-7].

We used data from the Global School Based Health Survey (GSHS) from Barinas-Venezuela, to estimate the prevalence of physical fighting and assess the strength of the association between this outcome and a selected list of explanatory variables. These explanatory variables were sex; having been bullied, parental supervision; age and substance use and were identified from the literature [2-7], as being associated with fighting. Information about correlates of physical violence may influence the design, implementation and evaluation of interventions aimed at reducing inter-personal violence among adolescents.

## Methods

### Study design and questionnaire administration

Our study involved secondary analysis of the 2003 Venezuela (Barinas) Global School-Based Health Survey (GSHS). The 2003 Venezuela GSHS was a school-based survey of students in grades 7^th ^to 9^th^. These grades were selected as they comprised the majority of 13-15 year olds in the country. The survey followed the standardized GSHS protocol that has previously been described elsewhere [8-10]. In brief however, potential participant identification involved a two-stage cluster sample design. This was used to produce data representative of all students in grades 7^th ^to 9^th ^in the study area.

At the first stage, schools were selected with probability proportional to enrollment sizes expected to have students between 13 and 15 years. At the second stage, classes were randomly selected and all students in selected classes were eligible to participate. Students who were not present on the day the survey administration in a particular school were not replaced and they were also not followed up. The overall response rate was 85%; resulting from a school response rate of 96% and a student response rate of 88%. A total of 2,249 students participated in the Barinas GSHS. Students self-reported their responses to each question in a multiple choice format questionnaire on a computer scannable answer sheet.

### Data Analysis

Data were analyzed using SUDAAN software, version (Research Triangle Park, North Carolina, United States). Frequencies and percentages for the outcome and explanatory variables were calculated. The questions from the questionnaire that were used were: During the past 12 months, how many times were you in a physical fight? Options ranged from 0 times to more than 12 times. For the main outcome, we re-coded the responses to the effect that having experienced a physical fight one or more times was re-assigned 1, otherwise 0. Another question was: During the past 30 days, how many days were you bullied? Students were allowed options 0 days up to all the 30 days. During the past 30 days, on how many days did you have at least one drink containing alcohol? During the past 30 days, how often did your parents or guardians really know what you were doing with your free time? The options were: Never; rarely; sometimes; most of the times, always. During your life, how many times have you used drugs éxtasis, cocaine, marijuana, or oler pega? Options available were 0 times to more than 10 times. In all the questions and their responses, if a student reported any number of times greater than 0 or days exposed to the behavior, the response was recorded as 1 (yes); otherwise 0 or missing as may have been appropriate.

In order to assess the association between any of the explanatory variables and the outcome (physical fighting), logistic regression analysis was conducted. Initially, bivariate model of the form: logit (physical fighting=1) = f (explanatory variable). The results are presented as unadjusted odds ratios. Further, multivariate models were run to assess the independent effect of bullying victimization on physical fighting. Backward elimination of variables was conducted to arrive at a parsimonious model. Confounders were: age, gender, substance use (alcohol drinking, or drug use), and parental supervision. The results of multivariate logistic regression analysis are presented as adjusted odds ratios (AORs).

## Results

Table 1 presents selected characteristics of the study sample of 2,229 Venezuelan adolescents in Barinas (median age 11-13 years old). Most of the sample was female (53.5%), 11-13 years old (57.8%), were not substance users, i.e., did not take alcohol or drugs (68.5%) and were without parental supervision during their free time (52.7%). Overall, 31.5% (37.0% males and 27.0% females) reported that they had been bullied in the past 30 days and 31.1% (47.4% males and 17.0% females) were involved in a physical fight in the last 12 months.

**Table 1:** Socio-demographic characteristics of the study population among Venezuela adolescents, 2003

	Total 100% (n = 2,229)	Males 46.5% (n = 1,061)	Females 53.5% (n = 1,168)
**Age (years):**			
≤ 13	57.8 (1,221)	55.4 (551)	59.9 (670)
14	24.0 (564)	24.0 (551)	24.0 (295)
15	12.8 (307)	14.4 (168)	11.1 (139)
≥ 16	5.4 (137)	6.2 (73)	5.0 (64)
**Substance use (alcohol or drug use**			
No	67.9 (1293)	62.0 (547)	72.6 (746)
Yes	32.1 (662)	38.0 (355)	27.4 (307)
**Parental supervision:**			
No	52.7 (1172)	53.6 (563)	52.0 (609)
Yes	47.3 (1057)	46.4 (498)	48.0 (559)
**Bullying victimization (number of days bullied/month)**			
None	68.5 (1361)	63.0 (584)	73.1 (777)
1-2	20.0 (404)	23.0 (210)	17.4 (194)
3-5	5.3 (107)	7.0 (62)	4.2 (45)
6-9	2.4 (50)	3.6 (34)	1.4 (16
10-30	4.0 (87)	4.2 (42)	3.9 (45)
**Fighting:**			
No	68.9 (1532)	52.3 (559)	83.0 (973)
Yes	31.1 (695)	47.4 (500)	17.0 (195)

Table 2 indicates that subjects who reported substance use (alcohol or drug use) were more likely to be in a physical fight than non-substance users (OR = 2.26; 95% CI [1.70, 2.99] for males and OR = 3.19; 95% CI [2.24, 4.53] for females). Bullying victimization was positively associated with physical fighting for both males and females. Parental supervision was negatively associated with physical fighting (0.57; 95% CI [0.44, 0.73] for males and OR = 0.51; 95% CI [0.37, 0.70] for females). Male respondents were more than four times likely to report physical fighting than females (OR = 4.41; 95% CI [3.61, 5.38]).

**Table 2:** Factors associated with physical fighting among Venezuelan adolescents in 2003

	Unadjusted odds ratios with 95% CI		
	Total	Males	Females
Age (years):			
≤ 13	1.00	1.00	1.00
14	0.98 [0.78, 1.22]	0.83 [0.62, 1.13]	1.14 [0.78, 1.66]
15	1.05 [0.80, 1.38]	0.81 [0.57, 1.15]	1.20 [0.73, 1.96]
≥ 16	1.28 [0.88, 1.85]	0.90 [0.54, 1.48]	1.93 [1.06, 3.52]
Gender:			
Female	1.00	-	-
Male	4.41 [3.61, 5.38]	-	-
Substance use (alcohol or drug use):			
No	1.00	1.00	1.00
Yes	2.80 [2.28, 3.45]	2.26 [1.70, 2.99]	3.19 [2.24, 4.53]
Parental supervision:			
No	1.00	1.00	1.00
Yes	0.56 [0.47, 0.68]	0.57 [0.44, 0.73]	0.51 [0.37, 0.70]
Bullying victimization (number of days bullied/month)			
None	1.00	1.00	1.00
1-2	2.93 [2.30, 3.73]	3.03 [2.06, 4.25]	2.57 [1.70, 3.87]
3-5	5.96 [3.92, 9.08]	6.01 [3.27, 11.07]	5.48 [2.87, 10.46]
6-9	8.55 [4.53, 16.14]	12.12 [4.55, 32.28]	3.92 [1.38, 11.15]
10-30	3.79 [2.42, 5.91]	3.63 [1.86, 7.08]	4.58 [2.36, 8.87]

Table 3 which reports results of the multivariable logistic regression analysis indicates that there was a dose-response relationship between bullying victimization and physical fighting (p-trend < 0.001). Compared to subjects who were not bullied, those who reported being bullied were more likely to engage in physical fighting after controlling for age, gender, substance use (smoking, alcohol drinking or drug use), and parental supervision (OR = 2.41; 95% CI [1.80, 3.23] for 1-2 days of bullying victimization per month, OR = 5.05; 95% CI [3.12, 8.18] for 3-5 days/month, OR = 7.46; 95% CI [3.62, 15.35] for 6-9 days/month, and OR = 3.80; 95% CI [2.28, 6.32] for 10-30 days/month).

**Table 3:** Association between bullying victimization and physical fighting among Venezuelan adolescents, 2003

Variable	*Adjusted odds ratios with 95% CI
Bullying victimization (number of days bullied in the last month):	
None	1.00
1-2	2.41 [1.80, 3.23]
3-5	5.05 [3.12, 8.18]
6-9	7.46 [3.62, 15.35]
10-30	3.80 [2.28, 6.32]

*Adjustments made for age, gender, substance use (alcohol drinking, or drug use), and parental supervision

## Discussion

Using the Global School Health Survey, we found a prevalence of having engaged in a physical fight in the past 12 months at 31.5% among adolescents in grades 7^th ^to 9^th ^in Barinas, Venezuela. Boys were more than twice as likely to report history of been engaged in a physical fight than girls. This male predominance in physical fighting has also been reported in other settings [11,12]. It is generally believed that societal tolerance toward male fighting may be responsible for the observed differences between the sexes [11,12].

Brown and Tappan [13], exploring ideas by Connell [14] have reported how adolescent girls' violence can be perceived as "hegemonic masculinity" [13]. In general, many studies have reported males exhibiting unhealthy behaviors than females. Granero et al [8] have reported on adolescents in Lara, Venezuela in which males were more likely to have engaged in sexual intercourse.

In bivariate logistic regression analysis, we found that physical fighting was positively associated with history of bullying victimization (being a victim of bullying), substance use, male sex, and being 15 years of age or older. These findings are similar to previous reports on adolescents physical fighting [5-7].

Finally we run multivariate (logistic) models to assess the independent effect of bullying victimization on physical fighting. We found that history of bullying victimization was positively associated with having engaged in a fight. Furthermore the effect was dose-dependent i.e. increased exposure to victimization was associated with high odds of having engaged in a fight.

Despite the fact that this study enrolled a large sample size and that the findings may be considered representative of the school-going age group in Barinas, Venezuela, there are a number of limitations that we must also highlight.

Furthermore, as we have reported results from data among adolescents in Barinas, the findings may only be representative of the adolescents in this area, and may not be representative of the rest of the country. In addition, data were collected via self reports. To the extent that the adolescents may have mis-reported, either intentionally or unintentionally, bias may have been introduced. Furthermore, data were collected in a cross sectional survey. One important limitation of a cross sectional study is the inability to determine causation. We cannot therefore confirm whether bullying victimization caused fighting or the variables are merely associated. However it is plausible to consider that some adolescents who may have been bullied may have engaged in fighting as a means of self-defense. Adolescents may have resorted to fighting to protect themselves from further inconsiderate behaviors from their peers. Nansell et al [15] have reported that adolescents who were bullied were more likely to bear weapons at school compared to those who had not so exposed. If there was a cause-effect relationship, this would possibly occur if the adolescents thought they needed to defend themselves. As is the case with all cross sectional data collection methods, we cannot ascertain whether any of the variables found associated with the outcome (physical fighting) are causal [16,17].

It can also be observed that the variables were assessed without consideration of their temporal sequence i.e. data of physical fighting related to 12 months prior to the study while most of the other variables were assessed for a much shorter period. The lesson however remains that having been involved in a physical fight within the past 12 months was associated bullying victimization, substance use, and limited or no parental supervision.

In a survey where adolescents are being asked potentially sensitive information such as substance use, physical violence and parental behaviors, there is always a concern as to whether their reports and the subsequent findings are reliable. We have no further information as to how reliable these findings are. However, previous studies from the United States using similar questions found that data had high reliability scores [18,19]. We are not aware of the reliability of the Global School Health Survey questionnaire among Venezuelan adolescent population. We suggest that future studies should be conducted to assess the reliability of, not only the GSHS questionnaire, but also other questionnaire data collection methods whose reliability outside of the United States, is not known. The Global Youth Tobacco Survey (GYTS) is one such questionnaire survey tool that may benefit from such reliability testing in a diversity of settings in low-, middle and high-income nations. Although the GSHS has some data that we used to estimate the prevalence and correlates of physical violence, the data set does not collect data on weapons bearing, neighborhood factors and household socio-economic position, experience of violence in the home; all factors having been previously found to be associated with adolescent violent behaviors [20-23].

## Conclusion

In a study of Venezuelan in-school students we found that physical fighting and bullying victimization was common among in-school adolescents in Barinas, Venezuela. The fact that victims of bullying were more likely to have engaged in physical fighting may be evidence supporting the notion that "violence begets more violence".

## Competing interests

The authors declare that they have no competing interests.

## Authors' contributions

ASM assembled the research team, participated in the interpretation of the results and led the manuscript drafting effort. PH participated in the interpretation of the results and drafting of the manuscript. SS participated in the interpretation of the results and drafting of the manuscript. ER conducted the data analysis, participated in the interpretation of the results and drafting of the manuscript. All authors read and approved the final manuscript.
